# Acute Effects of Foam Rolling on Hamstrings After Half-Marathon: A Muscle Functional Magnetic Resonance Imaging Study

**DOI:** 10.3389/fphys.2021.723092

**Published:** 2021-10-06

**Authors:** Dingbo Shu, Chuan Zhang, Siyu Dai, Shubo Wang, Jie Liu, Jianping Ding

**Affiliations:** ^1^Department of Radiology, Affiliated Hospital of Hangzhou Normal University, Hangzhou, China; ^2^School of Clinical Medicine, Hangzhou Normal University, Hangzhou, China; ^3^Institute of Sport Medicine, Hangzhou Normal University, Hangzhou, China; ^4^School of Physical Education and Sports, Central China Normal University, Wuhan, China; ^5^Department of Mechanical Engineering, University of Delaware, Newark, DE, United States

**Keywords:** recreational marathon runners, microvascular perfusion, intravoxel incoherent motion, inflammatory edema, T2 mapping

## Abstract

**Purpose:** Foam rolling (FR) is widely used for post-exercise muscle recovery; yet, the effects of FR on skeletal muscle inflammation and microvascular perfusion following prolonged exercise are poorly understood. We aim to address the gap in knowledge by using magnetic resonance imaging (MRI) T2 mapping and intravoxel incoherent motion (IVIM) sequences to study the acute effects of FR on hamstrings following half-marathon running in recreational runners.

**Methods:** Sixteen healthy recreational marathon runners were recruited. After half-marathon running, FR was performed on the hamstrings on the dominant side, while the other limb served as a control. MRI T2 and IVIM scans were performed bilaterally at baseline (pre-run), 2–3 h after running (post-run), immediately after FR (post-FR0), 30 min after FR (post-FR30) and 60 min after FR (post-FR60). T2, a marker for inflammatory edema, as well as IVIM microvascular perfusion fraction index *f* for biceps femoris long head (BFL), semitendinosus (ST) and semimembranosus (SM) were determined. Total Quality Recovery (TQR) scale score was also collected.

**Results:** Both T2 and *f* were higher at post-run compared to pre-run in all hamstrings on both sides (all *p* < 0.05; all *d* > 1.0). For the FR side, T2 decreased, and *f* increased significantly at post-FR0 and post-FR30 compared to post-run in all muscles (*p* < 0.05; all *d* > 0.4) except for *f* at BFL and SM at post-FR30 (both *p* > 0.05), though *f* at BFL was still marginally elevated at post-FR30 (*p* = 0.074, *d* = 0.91). Both parameters for all muscles returned to post-run level at post-FR60 (all *p* > 0.05; all *d* < 0.4) except for T2 at SM (*p* = 0.037). In contrast, most MRI parameters were not changed at post-FR0, post-FR30 and post-FR60 compared to post-run for the control side (*p* < 0.05; *d* < 0.2). TQR scores were elevated at post-FR0 and post-FR30 compared to post-run (both *p* < 0.05; both *d* > 1.0), and returned to the post-run level at post-FR60 (*p* > 0.99; *d* = 0.09). Changes in TQR scores compared to post-run at any time points after FR were correlated to T2 for ST at post-FR30 (*r* = 0.50, *p* = 0.047) but not T2 for other muscles and any changes in *f* values.

**Conclusions:** Hamstrings inflammatory edema and microvascular perfusion were elevated following half-marathon running, which were detectable with MRI T2 mapping and IVIM sequences. FR resulted in acute alleviation in inflammation and greater microvascular perfusion; however, the effects seemed to last only for a short period of time (30–60 min). FR can provide short-term benefits to skeletal muscle after prolonged running.

## Introduction

Running is a popular sport activity which can provide physical and psychological benefits to the runners. However, long-distance running induced muscular changes may also contribute to lower extremity musculoskeletal injury risks. Prolonged running has been reported to induce muscular strength decrease, plasma creatine kinase elevation, as well as delayed onset muscle soreness (DOMS) and feeling of fatigue (Hill et al., 2014; Clifford et al., 2016; Takayama et al., 2017), and may cause acute and chronic imbalance on multiple aspects of the human body (Junker and Stöggl, 2019). Repetitive overuse of muscles could lead to microdamage (Hooijmans et al., 2020), which triggers inflammatory responses, and the accumulative effects may ultimately result in muscle injury and dysfunction (Curran et al., 2008; Wan and Shan, 2016).

Greater microvascular perfusion in the hamstrings than the quadriceps was observed following running (Jungmann et al., 2019), suggesting the great involvement of the hamstrings for running activities. Hamstrings are consisted of biarticular muscles biceps femoris long head (BFL), semitendinosus (ST), and semimembranosus (SM), and play an important role in both knee and hip joints movements. Repeated eccentric contractions associated with running could lead to the accumulation of hamstrings microdamage, which puts hamstrings at risk for strain injury (Fyfe et al., 2013). Interestingly, Higashihara et al. (2020) showed that compared to the proximal region, the distal and middle portion of the hamstrings are more vulnerable to marathon-induced injuries. Therefore, post-running recovery methods that are effective in reducing local muscle damage at the middle and distal portion of the hamstrings could be promising remedies to prevent running related hamstrings injuries.

Foam rolling (FR) is a simple, time-efficient and effective post-exercise recovery tool that is able to reduce the risk of sports injury by shortening the recovery period with less negative impact on performance (Škarabot et al., 2015; Su et al., 2016; Jo et al., 2018; Behm and Wilke, 2019; Hodgson et al., 2019). This technique requires individuals to put their own body weight on a foam roller to exert pressure on the soft tissues, and roll back and forth starting at the proximal portion of the muscle (MacDonald et al., 2013). Evidence suggests that FR has a number of physiological benefits, including but not limited to increase blood flow, decrease tissue stiffness, promote muscle recovery, and reduce pain level (Aboodarda et al., 2015; Cavanaugh et al., 2016; Pablos et al., 2020; Phillips et al., 2021). However, whether FR can be used to reduce muscle damage induced by prolonged exercise, such as long-distance running, and therefore lower potential injury risks associated with such exercise modality remains to be addressed.

Therefore, the purpose of this study was to evaluate the acute effects of FR on hamstrings after a half-marathon in recreational runners using muscle functional magnetic resonance imaging (mfMRI). Specifically, we employed T2 sequence and intravoxel incoherent motion (IVIM) to assess inflammatory edema and microvascular perfusion respectively. T2 value reflects the level of muscle inflammation and is related to the severity of muscle injury (Maillard et al., 2004), and has been applied to detect muscle changes induced by exercise and monitor the recovery from muscle injury (Maillard et al., 2004; Higashihara et al., 2020; Hooijmans et al., 2020). MRI-IVIM model is a technique sensitive to the diffusion characteristics of water molecules, which can be used to evaluate the microvascular perfusion in human tissues, including the skeletal muscle (Iima and Le Bihan, 2016; Ogura et al., 2020). We hypothesized that the inflammation and microvascular perfusion as assessed using mfMRI at the mid portion of the hamstrings will both be elevated following half-marathon running, and that FR will result in decreased inflammation and increased microvascular perfusion.

## Materials and Methods

### Participants

A total of 16 recreational marathon runners (12 males) were recruited from the Zhejiang University Entrepreneurs Outdoor Association. This study received ethical approval (approval number 2021 (E2) - KS - 036) from the Affiliated Hospital of Hangzhou Normal University Research Ethics Committee, and informed consent was obtained from all participants prior to any data collection. Anthropometric characteristics for all participants are summarized in Table 1. Demographic and running related information were obtained from a questionnaire filled by the participant, including name, age, sex, monthly running distance and the pace during marathon. Recreational marathon runners were defined as runners who had not participated in formal distance running training and whose occupation was not running related. The main inclusion criteria were as follows: (1) history of regular long distance running for more than 1 year, (2) three or more regular weekly running sessions, (3) run for longer than 10 km at least once every week, (4) monthly running distance no less than 100 km, (5) having participated in a formal half-marathon or longer race at least once in the past year, and (6) no contraindications for MRI examinations. Those who have had musculoskeletal injuries at the lower extremities were excluded from the study.

**TABLE 1:** Participant and race characteristics of the 16 half-marathon runners.

	Age (years)	Weight (kg)	Height (cm)	BMI (kg/m^2^)	Monthly distance (km)	Speed (km/h)
Total (*n* = 16)	38.1 ± 4.5	64.4 ± 6.4	171.2 ± 5.2	22.0 ± 1.6	157.6 ± 50.2	11.1 ± 0.7
Males (*n* = 12)	38.7 ± 4.5	67.3 ± 3.4	173.2 ± 4.3	22.5 ± 1.2	162.5 ± 51.0	11.2 ± 0.6
Females (*n* = 4)	36.3 ± 4.8	54.5 ± 1.7	165.3 ± 1.7	20.0 ± 1.0	125.0 ± 25.2	10.4 ± 0.3

*Mean ± SD was used for descriptive statistics.*

*Monthly distance, monthly running volume in km as reported by the recreational runners; speed, average running speed during the half-marathon running.*

### Experimental Design

**FIGURE 1:**
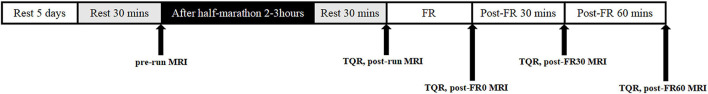
Example of the experimental protocol. FR, foam rolling; pre-run, baseline; post-run, 2–3 h after half-marathon running; post-FR0, immediately following FR; post-FR30, 30 min after FR; post-FR60, 30 min after FR; TQR, Total Quality Recovery scale; MRI, magnetic resonance imaging examination.

MRI data collections were performed five times for all participants, including baseline (pre-run), 2–3 h after half-marathon running (post-run), immediately after FR (post-FR0), 30 min after FR (post-FR30), and 60 min after FR (post-FR60). Participants lay on the MRI examination table for 30 min before the pre-run and post-run examinations, and were kept on the table throughout the rest of the experiment except for FR intervention. Half-marathon (21.0975 km) running was completed on the flat roads near our laboratory, and running related information, including average speed, time and distance were recorded using a phone app (Ledong Information Technology Co., Ltd., Chengdu, Sichuan). Subjects were asked to keep their RPE scale level below 15 throughout the half-marathon running. FR was performed immediately after the post-run MRI examination was finished. All participants were prohibited from running for long distance for at least 7 days, and refrained from performing any form of exercise for at least 5 days prior to the baseline MRI examination. Participants were asked to complete a Total Quality Recovery (TQR) scale before each MRI examination (except for baseline) to assess their overall sense of recovery (Kenttä, 1996; Kenttä and Hassmén, 1998), and one score was obtained at each time point to indicate the general sense of recovery for the participant. This is a scale ranging from 6 to 20 that was designed to assess the degree of fatigue recovery after exercise, with higher values indicating better sense of recovery. A full experimental protocol is shown in Figure 1.

### Foam Rolling Protocol

An experienced researcher (DS) showed all participants about the FR protocol prior to the first MRI data collection, and actual FR was performed after the post-run MRI examination was finished. A foam roller (LiNing, Shanghai, China; size 33 × 14 cm) constructed of a hollow polyvinylchloride pipe and surrounded by ethylene vinyl acetate copolymer was used for all FR massage in the current study. FR was performed on the dominant side for all participants, and the other side served as a control. To roll the hamstrings, the foam roller was placed on the ground, and the participant sat on the foam roller with hands placed on the ground to support their body weight. Participant was instructed to place as much of his/her weight as possible onto the foam roller and to roll gradually and continuously down from the hip to the knee. Each roll was completed in 5 s and the speed was controlled using a metronome. Once the foam roller reached the distal thigh, the participants were instructed to return the foam roller to the starting position (hip). The FR massage protocol was 60 s each with 5 repetitions. A 30 s rest interval was given between two consecutive FR massages, and pressure was controlled at 6–7 on visual analog scale (VAS) score (Aboodarda et al., 2015; Behm et al., 2020). VAS is a numeric scale ranging from 0 to 10 that measures the perceived pain rating, with 0 being no pain at all, and 10 being the worst pain imaginable. During the massage process, the same researcher (DS) assisted the participants to complete the FR as needed. All FR activities were performed at the MRI examination suite.

### Muscle Functional Magnetic Resonance Imaging

T2 mapping and IVIM sequences were employed to study the inflammatory edema and microvascular perfusion changes of the hamstrings in responses to half-marathon running and FR. The main perfusion indexes from IVIM sequences are perfusion fraction “*f*” and pseudo diffusion coefficient “D*.” However, D* value is highly influenced by noise, and accurately obtaining D* value requires high-quality data and long scanning time (Mastropietro et al., 2018), which may not be possible in post-exercise recovery time course studies that only permits short scanning times between consecutive scan sessions. In contrast, *f* value can be obtained accurately with relatively low-quality images and shorter scanning time. Therefore, *f* value was chosen to indicate microvascular perfusion from IVIM sequence in the current study. A 1.5 T MRI scanner (Magnetom Avanto, Siemens Healthcare, Germany) with an 18-channel body coil was used for all MRI data collection. Participants lay supine with legs fully extended within the coils. Sponges were added to the gaps in the coil in order to prevent movement artifact. T1 and T2 weighted image sequences were collected bilaterally from the greater trochanter to the medial condyle of the femur. T1-weighted anatomical scans in axial plane (*TR* = 850 ms, *TE* = 12 ms, FOV = 400 mm, thickness = 3.5 mm, average = 2, Bandwidth = 182 Hz/Px; scan time 2 min 34 s) were first collected. T1 sequence was collected to provide anatomical reference for IVIM images. T2 mapping sequence (*TR* = 2,000 ms, *TE* = 25, 50, 75, and 100 ms, FOV = 400 mm; thickness = 10 mm; average = 1; Bandwidth = 160 Hz/Px; scan time 3 min) was also collected. IVIM sequence (*TR* = 5,300 ms, *TE* = 78 ms, FOV = 380 mm, thickness = 3.5 mm, total slice number = 25, Bandwidth = 1,044 Hz/Px; *b* values [average = 0 (1), 10 (1), 20 (1), 40 (1), 80 (1), 110 (1), 140 (1), 170 (2), 200 (2), 300 (2), 400 (2), 500 (2), 800 (3) s/mm^2^; scan time 5 min and 20 s] in 3 orthogonal directions were subsequently collected in axial plane. Note that the slice thickness for T1 and IVIM sequences was set at 3.5 mm and was 10 mm for T2 mapping. The selection of different slice thicknesses was done to ensure image quality while keep the scan time short.

### Image Analysis

**FIGURE 2:**
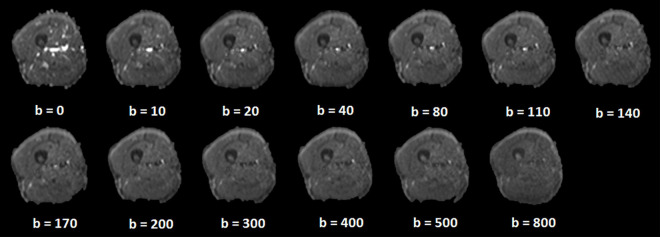
Example of intravoxel incoherent motion images at different b values.

For all participants, the mid-point line for the thigh region covered by the scans (from the greater trochanter to the medial condyle of the femur) was first identified and used as the anatomical reference (Higashihara et al., 2020). Four most adjacent slices to the mid-point line (2 above and 2 below) from the T2 sequence and 12 most adjacent slices to the line (6 above and 6 below) from the IVIM sequence were further processed. Therefore, the processed T2 (total thickness: 40 mm) and IVIM images (total thickness: 42 mm) represent approximately the same region at the mid-thigh. Example for IVIM images is shown in Figure 2.

**FIGURE 3:**
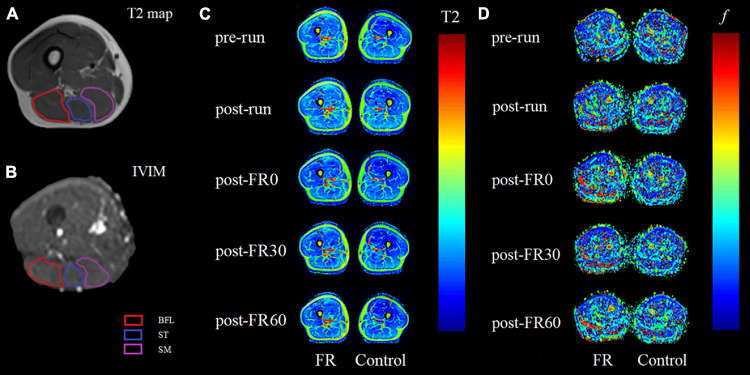
Example magnetic resonance images for raw T2 **(A)** and raw intravoxel incoherent motion (IVIM) b0 images **(B)** images with region of interest outlined for biceps femoris long head (BFL), semitendinosus (ST) and semimembranosus (SM). Panels **(C,D)** are color-coded maps for T2 mapping and IVIM *f* at different time points, respectively, with images on the left side being the limb that received foam rolling intervention. Light colors on the color-coded maps indicate lower values. FR, foam rolling; pre-run, baseline; post-run, 2–3 h after half-marathon running; post-FR0, immediately following FR intervention; post-FR30, 30 min after FR; post-FR60, 60 min after FR.

For T2 images processing, hamstrings, including BFL, ST, and SM were manually segmented on the selected T2-weighted images using syngo MR Workplace (syngo MR E11, Siemens, Germany). Care was taken to avoid the inclusion of any non-contractile tissues. The obtained T2 relaxation times were averaged over the 4 selected slices for each muscle and reported as the T2 value for each participant. Example for image segmentation (A) and color-coded T2 maps at different time points (C) are shown in Figure 3.

For IVIM images processing, a free open-source software platform Start MITK Diffusion (Division of Medical Image Computing, German Cancer Research Center) was used. Regions of interest corresponding to BFL, ST, and SM were manually segmented on b0 images, with the visual aid using T1-weighted anatomical images as reference when needed, and care was taken to avoid the inclusion of any non-contractile tissues. Pixels in which the fitting algorithm failed (*f* lower than 10^–4^) were excluded from the analysis (Mastropietro et al., 2018). The obtained fitted *f* values in percentage were averaged over the 12 selected slices reported as the *f* value for each muscle. Example for image segmentation (B) and color-coded IVIM *f* map at different time points (D) are shown in Figure 3.

### Statistics

**FIGURE 4:**
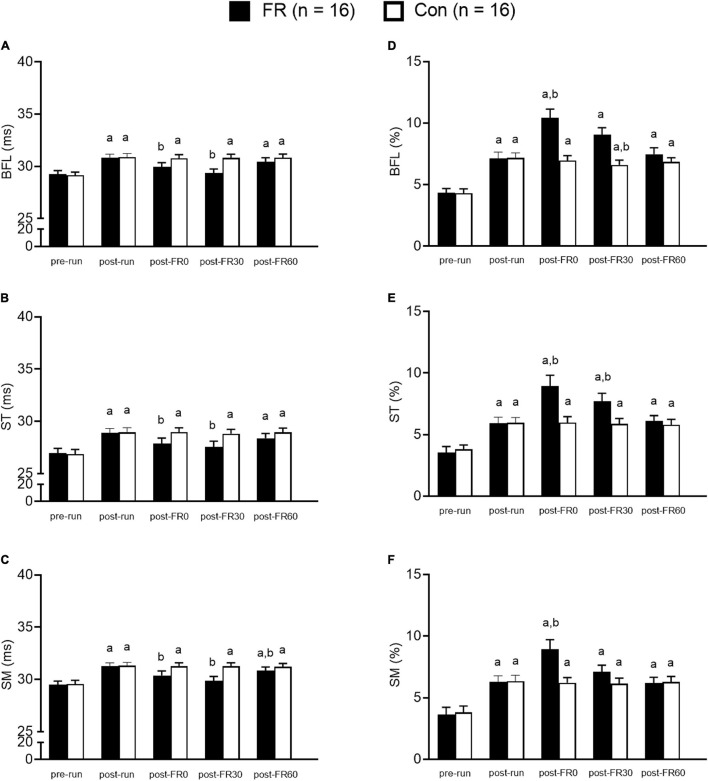
Bar graphs showing muscle functional magnetic resonance imaging results for foam rolling (FR) side and control (CON) side for the hamstring muscles at five different time points. **(A–C)** The T2 value changes from T2 mapping sequence. Panels **(D–F)** show *f* value changes from intravoxel incoherent motion sequence. BFL, biceps femoris long head; ST, semitendinosus; SM, semimembranosus; pre-run, baseline; post-run, 2–3 h after half-marathon running; post-FR0, immediately following FR intervention; post-FR30, 30 min after FR; post-FR60, 60 min after FR. ^a^Time effect for each single time point vs. pre-run within each group; ^b^time effect for each single time point vs. post-run within each group. Data are presented as Mean ± SE.

Statistical analyses were conducted using SPSS version 20.0 (IBM Corp., Armonk, NY). MRI parameters, including T2 and *f* values were analyzed using mixed-ANOVA, with sides (FR or control) as the between groups factor and time as the within groups factor. Bonferroni adjustment was applied for *post hoc* analysis as needed. Importantly, although we made all the pairwise comparisons, our primary interests for statistical analyses were (1) to compare the MRI parameters between the time points after half-marathon running vs. baseline, and (2) to compare the MRI parameters after FR vs. post-run, therefore the results were reported accordingly for the ease of interpretation. Results for the comparisons between each single time point vs. pre-run within each group, as well as between each single time point vs. post-run within each group are presented in Figure 4. TQR scores were also compared using one-way repeated ANOVA with time as the within subject factor, and Bonferroni *post hoc* adjustment was applied as needed. The relationship between changes in TQR scores and changes in MRI parameters (compared to post-run) at different post FR time points was examined using Pearson correlation. Statistical significance was set at *p* < 0.05. Mean ± SD was used for descriptive statistics. Cohen’s d (*d*) was calculated to indicate the effect size whenever applicable, with 0.2, 0.5, and 0.8 represent small, medium, or large effect size, respectively (Cohen, 2013). Post-examination power analysis was performed using G power and effect sizes were calculated for all muscles for T2 and f between post-run and post-FR0.

## Results

### Participants

All participants (12 men and 4 women) completed the half-marathon and all TQR and MRI examinations. Physical characteristics and running volume and speed for all participants are summarized in Table 1.

### Quantitative Muscle Functional Magnetic Resonance Imaging Parameters

Significant interactions were observed for T2 values and *f* values for all muscles (all *p* < 0.001).

*Post hoc* analysis revealed that T2 values were higher at post-run compared to pre-run for BFL, ST, and SM (all *p* < 0.001; all *d* > 1.0; Figure 4) for both FR side and control side. For the FR side, T2 values were lower at post-FR0 and post-FR30 compared to post-run for BFL, ST, and SM (all *p* < 0.05; all *d* > 0.5); T2 value was still significantly different at post-FR60 compared to post-run for SM (*p* = 0.037; *d* = 0.32); however, T2 value returned to post-run level at post-FR60 for BFL and ST (all *p* > 0.05; all *d* < 0.3). For the control side, T2 value was not significantly different at post-FR0, post-FR30 or post-FR60 compared to post-run for BFL, ST, and SM (all *p* > 0.05; all *d* < 0.1).

*Post hoc* analysis suggested that *f* values were higher at post-run compared to pre-run for BFL, ST, and SM (all *p* < 0.05; all *d* > 1.0; Figure 4) for both FR side and control side. For the FR side, *f* values were higher at post-FR0 compared to post-run for BFL, ST and SM (all *p* < 0.05; all *d* > 1.0), and was higher at post-FR30 compared to post-run for ST (*p* = 0.009; *d* = 0.77). *f* value was also higher at post-FR30 compared to post-run for BFL, although the result was only marginally significant (*p* = 0.074; *d* = 0.91). *f* value was not significantly different at post-FR30 compared to post-run for SM (*p* = 0.485; *d* = 0.4). *f* value returned to post-run level at post-FR60 in all three muscles (all *p* > 0.05; all *d* < 0.2). For the control side, *f* values were not significantly different at post-FR0, post-FR30 and post-FR60 compared to post-run for all three muscles (all *p* > 0.05; all *d* < 0.2), except for post-FR30 for BFL, which was slightly lower than post-run (*p* = 0.003; *d* = 0.35).

TQR scores were higher at post-FR0 and post-FR30 compared to post-run (15.06 ± 0.44 and 13.38 ± 0.50 vs. 12.63 ± 0.72, both *p* < 0.001, both *d* > 1.0). TQR score was not significantly different at post-FR60 compared to post-run (12.69 ± 0.6 vs. 12.63 ± 0.72, *p* > 0.99, *d* = 0.09). Changes in TQR scores compared to post-run at any time points after FR were not related to changes in *f* values or T2 values for any muscles (all *p* > 0.05), except for T2 for ST at post-FR30 (*r* = 0.50, *p* = 0.047). Post-examination power analysis suggested that we had high statistical power for this study (>0.81 for both T2 and f for all three muscles).

## Discussion

To the best of our knowledge, this is the first study that employed mfMRI to evaluate the efficacy of recovery strategies on skeletal muscle recovery following prolonged running. In this study, MRI T2 mapping and IVIM sequences were used to non-invasively evaluate inflammatory edema and microvascular perfusion in hamstrings before and after FR. The microscopic physiological changes of BFL, ST and SM at five time points of pre-run, post-run, post-FR0, post-FR30, and post-FR60 were demonstrated bilaterally in recreational runners. After half-marathon, T2 and *f* values for all hamstrings were increased compared to pre-run on both sides. After FR (post-FR0), T2 values decreased while *f* values increased compared to post-run for BFL, ST muscle, and SM muscle on the FR side, with minimal changes observed on the control side. TQR score was significantly increased, indicating improved sense of recovery following FR. All changes on *f* values on the FR side and TQR returned to post-run level at post-FR60, indicating that the effect of FR is acute and lasts approximately 30–60 min following one session of FR. Our data suggest that FR massage protocol employed in the current study acutely reduced hamstrings inflammation and increased microvascular perfusion after the running.

### After Half-Marathon

We examined the participants shortly after (2–3 h) the half-marathon running and found that T2 values for all three muscles on both sides were significantly higher than pre-run, which is consistent with findings from previous studies that investigated T2 changes in skeletal muscle following running (Higashihara et al., 2020; Hooijmans et al., 2020). In addition, *f* values for all hamstrings were higher compared to pre-run, which is consistent with a previous study that found increased *f* value in the skeletal muscle after running (Jungmann et al., 2019). These changes in MRI signals may be caused by the loss of calcium homeostasis, the pro-inflammatory response, as well as the increased oxidative stress after long-distance running (Siegel et al., 2001; McAnulty et al., 2003; Overgaard et al., 2004; Hooijmans et al., 2020). Therefore, our results suggest that MRI T2 and IVIM sequences can be used to monitor the microscopic changes in the muscle, particularly to evaluate the presence of inflammatory edema and microdamage after exercise.

### After Foam Rolling

We found that T2 values for BFL, ST, and SM muscles were significantly lower compared to post-run immediately following acute FR intervention for the FR side. In contrast, no significant changes were observed for the control side for any muscles. The likely explanation for the decreased post-FR0 T2 value is the alleviation of inflammatory edema. Myofascia, including blood and lymph, has close interaction with surrounding tissues (Barnes, 1997). An animal study suggested that the water content of myofascia has a high adaptability to mechanical stimulation (Schleip et al., 2011). It is likely that the mechanical force exerted on the muscle during FR intervention caused a water content redistribution between muscle and the surrounding tissues, leading to decreased water volume retained within the muscle tissue and manifest as decreased T2 values. Our results indicate that FR can effectively reduce the level of edema in muscle tissue after long-distance running, which could be beneficial for muscle recovery.

We found that the *f* values for BFL, ST, and SM were significantly higher than post-run following acute FR intervention. In contrast, there were no significant changes in *f* for any of the hamstrings in the control group. The results indicate that the microvascular perfusion for BFL, ST, and SM was significantly increased after FR. This is consistent with results from a previous study which suggested that blood flow in the lateral femoral posterior artery was increased after FR (Hotfiel et al., 2017). Similarly, using near-infrared spectroscopy, Soares et al. (2020) found that FR could improve skeletal muscle oxygenation and microvascular reactivity. One possible explanation for the increased microvascular perfusion is that FR changes the viscoelasticity and thixotropy of the fascia, and these changes combined with the heat production and friction induced by FR, would result in increased blood flow in the muscle (Bradbury-Squires et al., 2015; Cheatham et al., 2015). In addition, the increased shear stress on the vascular wall caused by external compression through FR is a powerful stimulus for nitric oxide (NO) production (Okamoto et al., 2014), which is an vasoactive substance known to modulate microvascular function and therefore could lead to microvascular perfusion changes (Kurose et al., 1994; Paniagua et al., 2001). The enhanced microvascular blood flow, as well as greater oxygen delivery as induced by acute FR intervention, could stimulate the mitochondria to accelerate adenosine triphosphate and phosphocreatine filling, a process that contributes to the elimination of metabolic waste products during recovery (Romero-Moraleda et al., 2019). Therefore, the increased microvascular perfusion as a result of FR may play an important role in promoting the recovery of muscle microdamage after exercise.

Results of TQR scores suggested that the participants’ overall sense of recovery was improved immediately following FR. The improvement is likely due to the combination of both physiological and psychological factors. Physiologically, the increased microvascular perfusion after FR could accelerate the elimination of metabolic wastes (Cheatham et al., 2015; Romero-Moraleda et al., 2019). In addition, massage may facilitate the reconstitution of exercise induced Ca^2+^ -ATPase activity decrease (Ogai et al., 2008) and therefore reduce muscle stiffness and provide better sense of recovery. Psychologically, FR intervention modifies the perception of stretch (Krause et al., 2019), increases pressure pain threshold (Cheatham and Kolber, 2018) and improve mood (Arroyo-Morales et al., 2008), which could all contribute to the increased TQR scores in response to FR.

### *Thirty* and *Sixty* Minutes After Foam Rolling

At post-FR30, T2 values for BFL, ST, and SM for the FR side were still lower compared to post-run. *f* values for SM and BFL were still elevated at post-FR30 compared to post-run, but *f* value for SM returned to post-run level. In contrast, both T2 and *f* values remained unchanged at post-FR30 compared to post-run and post-FR0 for the control side for all muscles, except for BFL. Sixty minutes after FR, T2 value for BFL returned to post-run level, while remained elevated for ST and SM compared to post-run. *f* values for BFL, ST, and SM all returned to post-run level at post-FR60. TQR scores were still elevated at post-FR30 and returned to post-run level at post-FR60. Our data, combined with data at post-FR0, indicate that (1) mfMRI is capable of detecting the physiological changes induced by FR, and (2) FR could provide short-term benefits to muscle recovery that generally lasts 30–60 min.

As previously stated, the increase in T2 value may be due to the increase of microvascular perfusion or muscle inflammatory edema after exercise (Esposito et al., 2013; Sharafi et al., 2017; Messer et al., 2018). In this study the lowest T2 for the FR side were observed at post-FR30 for all muscles, while the peak values for *f* were presented at post-FR0 for all muscles. Combing the pattern changes for both T2 and *f*, we postulate that FR is capable to both reduce inflammatory edema as well as improve microvascular circulation, with the later effect being more acute while persistently reduce edema for a relatively longer period. It is possible that the improvement in inflammatory edema and microcirculation could facilitate the microdamage recovery experienced by the hamstrings following half-marathon running, and therefore reduce future injury risks and shorten the recovery time needed to prepare for the next race; however, more studies with longer follow-up times are needed in order to confirm this inference. At post-FR60, T2 value remained lower than post-run for ST and SM, indicating that the effects of FR to reduce inflammatory edema could last for at least an hour for certain muscles. Studies that investigate the time course for FR to reduce muscle inflammatory edema are needed to determine if such effect could persist even longer than an hour. In addition, it is possible that more frequently applied FR during recovery could shorten the inflammation process through the removal of interstitial fluid, the dissolved proteins and lymphocytes *via* the lymphatic system, although clearly more research is needed to confirm this notion (Crane et al., 2012; Macdonald et al., 2014; Pablos et al., 2020).

Our results that microvascular perfusion was still elevated at post-FR30 for the FR side is consistent with a previous study that measured blood flow response to FR. Using Doppler ultrasound, Hotfiel et al. (2017) measured arterial perfusion after FR intervention at two time points (post-FR0 and post-FR30), and found that the increase in blood flow was more prominent at post-FR0 than post-FR30, but remains higher at post-FR30 compared to baseline. In addition, in our study we found that all *f* values return to post-run level at post-FR60. Therefore, it can be concluded that effects of FR to increase blood flow is somewhat transient (for about 30 min), and future studies that manipulate different variables of the FR protocols, such as rest interval or the number of repetitions, to determine whether more significant blood flow effects can be achieved are warranted.

Overall, changes in TQR scores were not significantly related to either MRI parameters for any of the three measured muscles. The lack of association between changes in TQR scores and MRI parameters may be attributed to several possibilities. First, since this is not the primary goal of this study and we only enrolled 16 participants, the relatively small sample size may not provide enough power to detect the true relationship between the two measurements. Second, underlying factors driving the improved TQR scores following FR may be multifaceted and cannot be specifically attributed to the improved microvascular perfusion or alleviated inflammatory edema as measured in the current study. It was previously reported that FR can induce endorphin release (Weerapong et al., 2005) and increase pain threshold (Cheatham and Kolber, 2018), which are probably important factors that could modulate participant’s overall sense of recovery. It should be noted that while the TQR scale used in this study only evaluates the overall sense of recovery for the participants, studies have demonstrated that unilateral FR can elicit crossover effect on the contralateral limb to alleviate pain (Aboodarda et al., 2015; Cavanaugh et al., 2017; Behm and Wilke, 2019). Thus future studies with larger sample size and sham group (light rolling) are needed to determine whether improvement in microvascular perfusion and inflammation are contributing factors to the better sense of recovery following FR.

To sum up, the use of FR after half-marathon could provide both physiological benefits as well as overall sense of recovery at least for a short period of time. In this study we only evaluated the effects of FR on hamstrings, but it can be inferred that FR may also be used to facilitate the acute recovery of other muscles following strenuous exercise. Importantly, it should be pointed out that FR should be avoided in case of acute muscle injury. External loading exerted through FR intervention may further stretch the injured muscle tissue.

### Limitation

This study has several limitations. First, as previously discussed, high quality images and longer scan time are required to accurately determine D*; however, due to the time constraint of our study protocol, obtaining high-quality images to calculate D* may not be feasible. Future studies with better image quality are needed to determine the D* changes following FR intervention. Second, TQR scale used in the current study is designed to assess the overall sense of recovery. Due to a lack of control group, it is possible that the increased TQR score following FR was partly due to passive recovery. However, this is unlikely to be the case, as TQR score returned to post-run level 60 min after FR. Third, we monitored the participants up to 60 min following FR, therefore it is unclear how the microscopic physiological changes observed in the current study can be translated into better longitudinal recovery for recreational runners. Future studies with longer follow-up periods are needed to determine whether FR provides long-term benefits for post-exercise recovery and injury prevention. Last but not the least, the relatively small number of participants precludes the possibility of gaining conclusive evidence regarding whether improvement in microvascular perfusion and inflammation are driving factors for the alleviated TQR scores. Studies with larger sample size and sham group (light rolling) are therefore needed before definitive conclusion can be drawn.

## Conclusion

Hamstrings inflammatory edema and microvascular perfusion were elevated following half-marathon running, which were detectable with mfMRI T2 mapping and IVIM sequences. FR resulted in acute alleviation in inflammation and greater microvascular perfusion, as well as improvement in overall sense of recovery; however, the effects only seemed to last for a short period of time (30–60 min). FR can provide short-term benefits to skeletal muscle after prolonged running. More research is needed to explore whether the short-term benefits seen in the current study can be translated into reduced injury risks and faster recovery.

## Data Availability Statement

The raw data supporting the conclusions of this article will be made available by the authors, without undue reservation.

## Ethics Statement

The studies involving human participants were reviewed and approved by the Affiliated Hospital of Hangzhou Normal University Research Ethics Committee. The patients/participants provided their written informed consent to participate in this study.

## Author Contributions

DS designed the study, performed data collection and data analysis, and drafted the initial manuscript. CZ designed the study and performed data analysis and interpretation. SD designed the study and assisted with data collection and data analysis. SW and JL assisted with data collection, analysis, and interpretation. JD designed the study, performed data interpretation and analysis, and contributed to the structure, planning, and execution for this project. All authors approved the final manuscript as submitted.

## Conflict of Interest

The authors declare that the research was conducted in the absence of any commercial or financial relationships that could be construed as a potential conflict of interest.

## Publisher’s Note

All claims expressed in this article are solely those of the authors and do not necessarily represent those of their affiliated organizations, or those of the publisher, the editors and the reviewers. Any product that may be evaluated in this article, or claim that may be made by its manufacturer, is not guaranteed or endorsed by the publisher.
